# Study of the effect of *in situ* minerals on the pyrolysis of oil shale in Fushun, China†

**DOI:** 10.1039/d2ra02822k

**Published:** 2022-07-13

**Authors:** Wang Xinmin, Wang Qing, Wu Chunlei

**Affiliations:** Engineering Research Centre of Oil Shale Comprehensive Utilization, Ministry of Education, Northeast Electric Power University Jilin City Jilin 132012 China rlx888@126.com

## Abstract

Herein, the effect of Fushun oil shale minerals on its kerogen has been investigated. The samples were obtained under non-isothermal conditions at different final temperatures. Scanning electron microscope (SEM) analysis revealed that the silicates were layered and the carbonates were tightly bound to each other. The combination of silicates and carbonates led to close combination of minerals and organic matter and the organic matter was contained in the minerals. The Brunner–Emmet–Teller (BET) experiments conclude that during the non-isothermal pyrolysis process, the specific surface area increased, and then, decreased, which proves the adsorption effect of silicates on oil shale pyrolysis products and the adsorption effect of carbonates was weak. The activation energy of four samples was calculated *via* Flynn–Wall–Ozawa (FWO) and Friedman kinetic analysis under different heating rates in a non-isothermal process, wherein the average activation energy of the sample containing silicate was 177.60 kJ mol^−1^ at minimum while that of carbonate was 250.45 kJ mol^−1^ at maximum, which proves that the catalytic promotion effect of silicate was greater than the inhibition effect of carbonate. The pyrolysis products obtained by Flash pyrolysis-gas chromatography-mass spectrometry (Py-GC/MS) under isothermal pyrolysis conditions were primarily composed of aliphatic hydrocarbon structures, which had different degrees of impact on the production of heteroatoms. This work provides a reliable theoretical basis for future studies on the influence of minerals on pyrolysis of organic matter in oil shale.

## Abstract 2

In this paper, the effect of *in situ* minerals on the pyrolysis of Fushun oil shale is studied by combining isothermal and non isothermal experiments.

## Graphical abstract

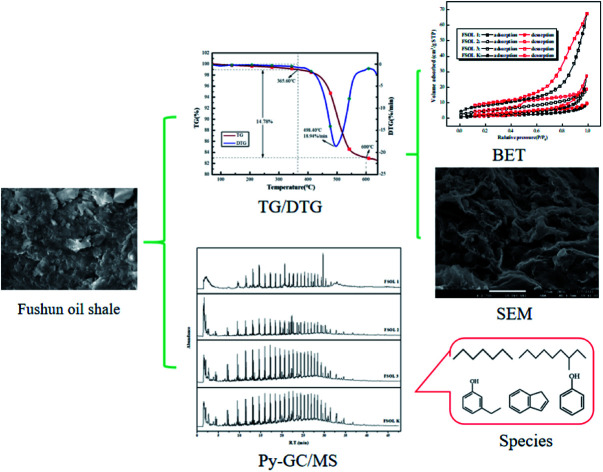

## 1 Introduction

Oil shale is considered a major alternative energy source to traditional fossil energy sources due to its abundant reserves and its ability to produce shale oil and gas through high temperature dry distillation technology.^1^ China is the largest producer of shale oil with 329.89 billion proven reserves, out of which the Fushun West Open Pit is the main oil shale producing area.^2^ Oil shale is a sedimentary rock consisting of inorganic minerals and organic matter kerogen, wherein the solid organic matter exists within the mineral skeleton, which plays a crucial role in the dry distillation and refining of oil shale due to high mineral content and close fugacity with organic matter.^3–6^ However, there are limited studies on the interaction between kerogen and minerals during the pyrolysis of oil shale. To investigate the role of minerals in the pyrolysis process of kerogen, herein, relevant experiments were performed on the oil shale, two demineralized substances, and kerogen under the same conditions. The relationship between organic matter kerogen and inorganic minerals was studied by comparing the experimental results.

Inorganic minerals (carbonates, silicates, and small amount of pyrite) are the main components of oil shale. There are physical and chemical methods for the removal of minerals, among which HCl–HF–HCl stepwise acid washing is considered as the most effective chemical extraction method for extracting kerogen that is widely used in kerogen production.^7–10^ The step-by-step acid washing of oil shale by HCl and HF acids can deliver samples containing different minerals.^11^ In recent years, several scholars have studied the effect of minerals on oil shale pyrolysis. Some of them have employed thermogravimetric analysis (TG) to compare the activation energy of oil shale and kerogen. A few researchers have suggested that the activation energy of kerogen is low, which indicates that the effect of minerals is inhibited.^12,13^ However, a few other scholars have concluded that the activation energy of kerogen is high, which signifies that minerals have a facilitating effect on the oil shale pyrolysis.^14,15^ Also, it has been shown that carbonates have a facilitative effect and silicates have an inhibitory effect.^16^ This could be because of different sample compositions of oil shale. Using SEM, BET, XRD, TG-FTIR, and Py-GC/MS, several scholars have conducted detailed study on the impact of minerals on kerogen.^17^ SEM and BET are important ways to characterize the pore structure of oil shale. Pores are usually classified into micropores (<2 nm), mesopores (2–50 nm), and macropores (>50 nm) according to their diameters.^18^ The pore structure can affect product release and semicoke composition due to the adsorption properties of minerals on its pyrolysis products.^19–21^ Borrego *et al.*^22^ employed thermogravimetric analysis, differential thermal analysis, and X-ray diffraction (XRD) experiments to assess the influence of amount and composition of mineral matter on thermal behavior of oil shales. Also, their impact on the amount of hydrocarbons released was evaluated, which demonstrated that minerals are responsible for the delayed hydrocarbon generation during heating process. Yan *et al.*^11^ applied TG-FTIR to investigate the catalytic effect of mineral matrix on the pyrolysis and combustion of kerogen. The results show the combined effect of all the minerals on the pyrolysis process of organic matter with a facilitating effect and on the oxidation of kerogen. Wang *et al.*^23^ studied the effect of carbonate and silicate in birch oil shale *via* aluminum retort pyrolysis, and concluded that carbonate can promote shale oil production and inhibit hydrocarbon gas production, while silicate can inhibit shale oil production and promote hydrocarbon gas production. Also, it was concluded that both the minerals can increase the degree of shale oil aromatization. Ballice *et al.*^24,25^ investigated the effect of own minerals on the generation of volatile hydrocarbons *via* pyrolysis of two Turkish oil shales. The group demonstrated that carbonates have a catalytic effect on the hydrocarbon generation, while silicates have an inhibitory effect on the same. Also, pyrite did not impact hydrocarbon generation. Ekstrom *et al.*^26^ found that stripping minerals reduced H_2_ precipitation from the pyrolysis of two australian oil shales, suggesting that minerals catalyze the breakdown of shale oil to H_2_. Py-GC/MS has been considered as an important research tool to study the pyrolysis products of oil shale. It can be used to better understand the distribution of gaseous products of Fushun oil shale and kerogen while avoiding the interference of low-temperature pyrolysis products. JR *et al.*^27^ explored the release pattern of volatile products from oil shale and casein using pyrolysis-gas chromatography. The results show that the pyrolysis of oil shale decreased the amount of long-chain hydrocarbons and increased the amount of short-chain and aromatic hydrocarbons, indicating that minerals can promote hydrocarbon cracking reactions and aromatization reactions.

The above studies illustrate the effect of minerals *via* different technical means to a certain extent, but they do not provide a detailed study on the effect of minerals in Fushun oil shale on kerogen. Therefore, herein, a detailed and systematic study of the structural composition and pyrolytic behavior of Fushun oil shale *in situ*, carbonate, silicate, and kerogen was conducted *via* XRD, TG-FTIR, SEM, BET, FTIR, and Py-GC/MS. The purpose of this work is to study the influence of minerals on the pyrolysis of dry distillation of kerogen and provide guidance to enhance the yield of oil and gas production from Fushun oil shale from the oil shale itself, which can reduce the cost and improve oil recovery.

## 2 Experimental

### 2.1 Sample preparation

Fushun oil shale samples were selected and extracted to prepared the kerogen as per the national standard of coal sample preparation method “GB/T 474-2008”. The collected samples were sealed and stored to avoid oxidation. The samples were initially crushed by crusher, then crushed by high-speed crusher, and sieved to below 180 mesh. Finally, the samples were mixed and dried at 40 °C in vacuum drying oven. Herein, we applied HCl (20%) to remove carbonate to prepare silicate containing samples. Application of HF (40%) to prepare carbonate-containing samples by removal of silicate. Application of HCl (20%)–HF (40%)–HCl (20%) to prepare caseinate, all acid-washed samples were repeatedly washed with deionised water until the filtered water was neutral. Finally, the original oil shale sample (FSOL1), silicate containing (FSOL2), carbonate containing sample (FSOL3), and kerogen (FSOLK) were obtained. Table 1 summarizes the data from the industrial analysis of Fushun oil shale.

**Table 1:** The proximate data of Fushun oil shale

Sample	Proximate analysis			
	Moisture	Volatile matter	Ash	Fixed carbon
Oil shale	2.3 ± 0.1	17.6 ± 0.2	78.8 ± 0.4	1.3 ± 0.1

### 2.2 XRD data

The XRD patterns of kerogen sample were recorded using Bruker D8 Venture XRD analyzer (Bruker, Germany) equipped with a Cu tube operating at 40 kV and 40 mA.

### 2.3 TG data

The equipment used for the TG-FTIR experiments consisted of a TGA/DSC I TG analyzer (Mettler-Toledo, Switzerland), and a NICOLETis 10 FTIR coupler (Nicolet, USA). The gas tightness of the whole reaction system was checked before the test. Using high purity nitrogen as a protector, the inert gas ensured that the test sample did not react with oxygen and it was easy to observe the change of sample mass under an inert atmosphere. A sample of about 10 mg was placed and heating was started with an initial starting temperature of 50 °C and termination temperature of 650 °C, at the heating rate of 20, 30, 40, and 50°C min^−1^.

### 2.4 Kinetic data

Based on the thermal analysis, the differential and integral forms of the gas–solid reaction rate are presented in eqn (1)–(3). Pyrolysis kinetics are derived from thermogravimetric data as follows:1
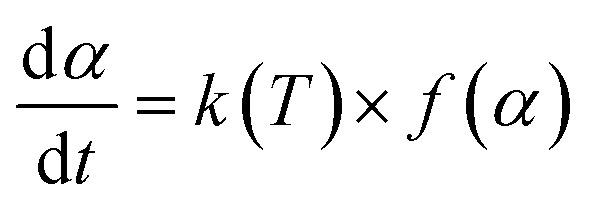
where *k*(*T*) is the reaction rate constant and *f*(*α*) is the reaction mechanism function, the normal form of *f*(*α*) is: *f*(*α*) = (1 − *α*)^*n*^. *T*, and *α* are the absolute temperature, reaction time, and reaction conversion rate, respectively.2
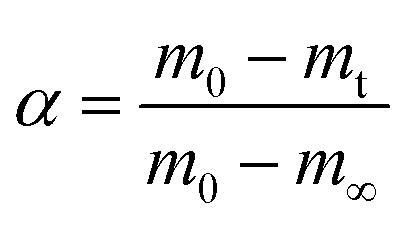
3
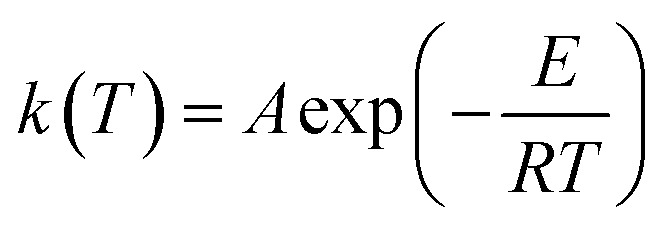
4
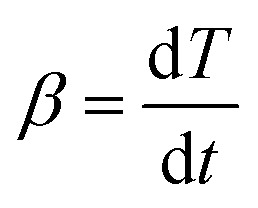
*m*_0_, *m*_t_, and *m*_∞_ are the original, actual and final masses, respectively; *A*, *E*, and *R* are the prefactor (S^−1^), activation energy (kJ mol^−1^), and universal gas constant (8.314 Jmol^−1^K).

Combining eqn (1)–(4) obtained the kinetic equation eqn (5):5
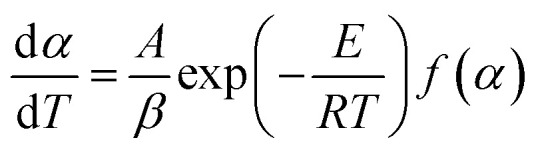


#### 2.4.1 Flynn–Wall–Ozawa (FWO) method

As per the integral expression (2), combining eqn (6) to establish eqn (7) by transforming. Then, the integral approximation of eqn (8) was used to obtain eqn (9).6
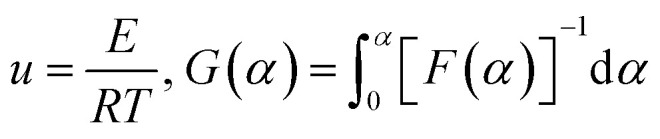
7
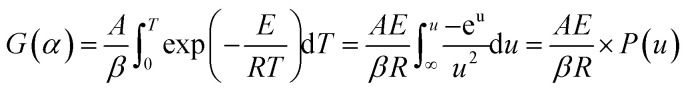
8
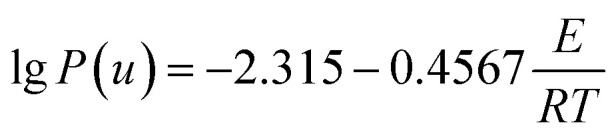
9
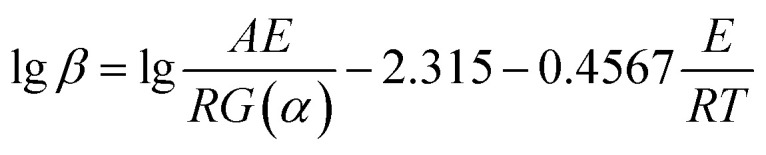


Eqn (9) is the mathematical expression of FWO method. *G*(*α*) is constant at same conversion rate. Hence, according to the data, the relationship between lg *β* and 1/*T* is linear for different heating rates at the same conversion rate. Then, the activation energy of the reaction was obtained from the slope. However, an approximation was taken for the solution of the temperature integral in the derivation of the above equation; hence, the solution of the activation energy can cause different degrees of deviation.^28,29^

#### 2.4.2 Friedman method

A deformation of both sides of eqn (4) and a logarithmic operation on the left and right sides gives the equation for Friedman's method [69].10
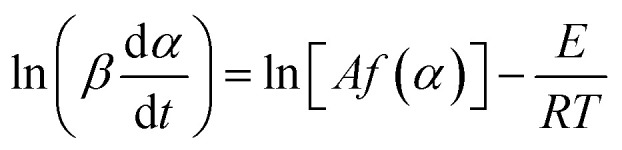


The activation energy at the same conversion rate and at different rates of warming can be calculated from the equation, the value of which can be obtained from the slope of ln(d*α*/d*T*) and 1/*T*.

### 2.5 Horizontal tube furnace method

**Fig. 1:**
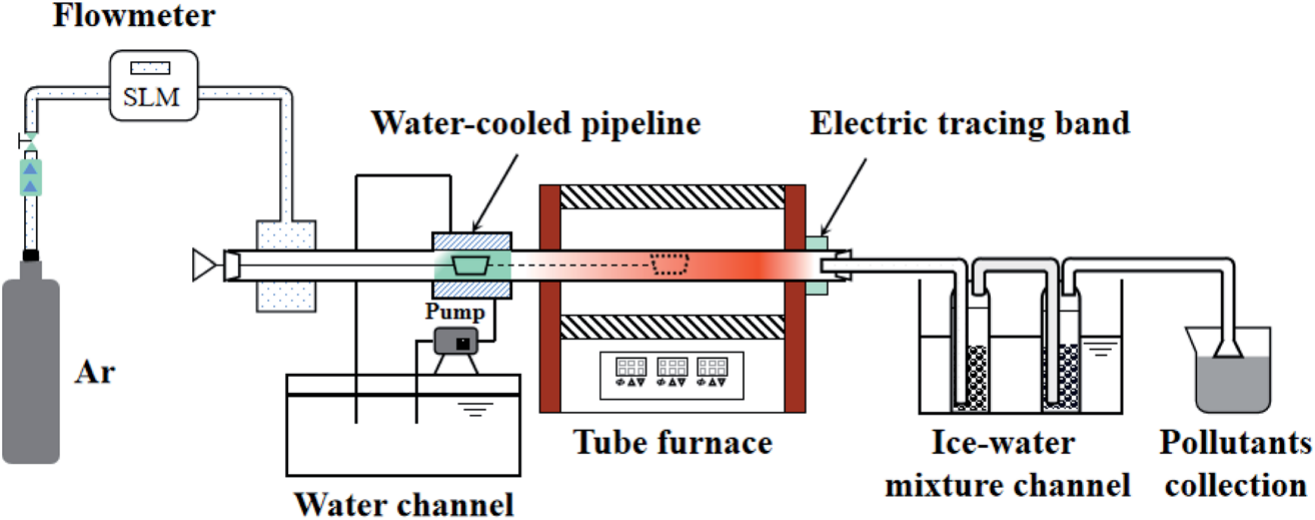
Schematic diagram of the horizontal tube furnace system.

The pyrolysis experiment was performed in an openable, high-temperature, fixed-bed experimental apparatus. The horizontal tube furnace enables samples to be prepared at different end temperatures and the samples prepared are used for SEM and BET tests to observe changes in the apparent morphology and pore structure of the pyrolysis products with temperature. Fig. 1 shows the schematic diagram of the horizontal tube furnace syste. The heater was controlled by three non-independent temperature control sections, using the one-to-two control method. The temperatures of the three heating zones were controlled by a main meter to ensure the consistency and overall stability of the temperature in each section of the reactor. The length of the constant temperature reaction zone was about 100 mm. The gas seal device at the gas inlet end was designed with a cavity sleeve, with small holes evenly arranged on the circumference of the inner cylinder. The gas entered the sleeve from the left and right sides, mixed in the cavity, and then, entered the reactor evenly along the circumference through the small holes. This set-up ensured gas seal, *i.e.*, the valve at the gas inlet end opened to send the ark into the furnace quickly without outside air entering the furnace to contaminate the inert atmosphere. 2 g of sample was placed in a quartz glass reactor. The initial temperature was 20 °C, and the temperature rise rate was 30°C min^−1^ to the final temperatures of 400, 470, and 550 °C to produce samples. The prepared samples were subjected to SEM and N_2_ adsorption experiments to observe the changes in pore size and apparent shape of the four Fushun samples at different end temperatures.

### 2.6 SEM data

Surface and morphology of Fushun oil shale samples were studied using a JSM-7610 thermal field emission scanning electron microscope from Japan Electronics Corporation at an accelerating voltage of 0.1–30 kV and Zoom:25-1 000 000. High stability submerged Schottky thermal field emission electron gun was employed along with a focusing mirror. 2-Stage electromagnetic lens convergence system was used and beam intensity could be continuously adjusted. The objective diaphragm was 4-stage adjustable with fine adjustable alignment in *X*/*Y* direction. The samples for the SEM application were FSOL 1, FSOL2, FSOL 3, FSOLK prepared by acid washing and four samples prepared by horizontal tube at different end temperatures of 400, 470 and 550 °C samples.

### 2.7 BET data

The specific surface area, pore volume, pore size distribution, surface energy, adsorption, and desorption characteristics of solid samples were determined *via* physical adsorption method using TriStar II 3020 specific surface area and porosity analyzer from Micromeritics, USA. The vacuum pump parameters were 230 V, 50/60 Hz, 180VA. The working gas pressure was 0.1–0.15 MPa; BET specific surface area analysis range was 0.001 m^2^ g^−1^ to no upper limit; pore size analysis range was 0.35 nm to 500 nm; adsorbent was N_2_; pretreatment temperature range was ambient temperature to 400 ± 5 °C; and input power was 200 VA.

### 2.8 Py–GC–MS data

The samples (5 mg,<0.2 mm) was placed in a multi-shot pyrolyzer (Frontier Laboratories EGA/PY-3030D) using a double-shot sampler, and then the samples pyrolysis products were directly used for GC-MS analysis (PerkinElmer SQ8 GC-MS) coupled with the pyrolyzer using Ar as the carrier gas. The chromatographic column is a HP-5 MS capillary (30 m × 0.25 mm × 0.25 μm). The qualitative and quantitative analysis of the spectra was done using TurboMass Ver 6.1.0 software and the search library was NIST 14. The relative contents of compounds fractions were calculated using the technique of area normalization. Pyrolysis condition: pyrolysis time = 10 s; pyrolyzer furnace temperature = 600 °C. GC-MS acquisition parameters: oven initial temperature 50 °C for 5 min; ramp rate 10°C min^−1^ to 280 °C; hold = 20 min; injection port temperature = 250 °C; transfer temperature = 250 °C; source temperature = 250 °C; scan range = 50 to 450 Da; diversion ratio = 50/1.

## 3 Results and discussion

### 3.1 XRD analysis

**Fig. 2:**
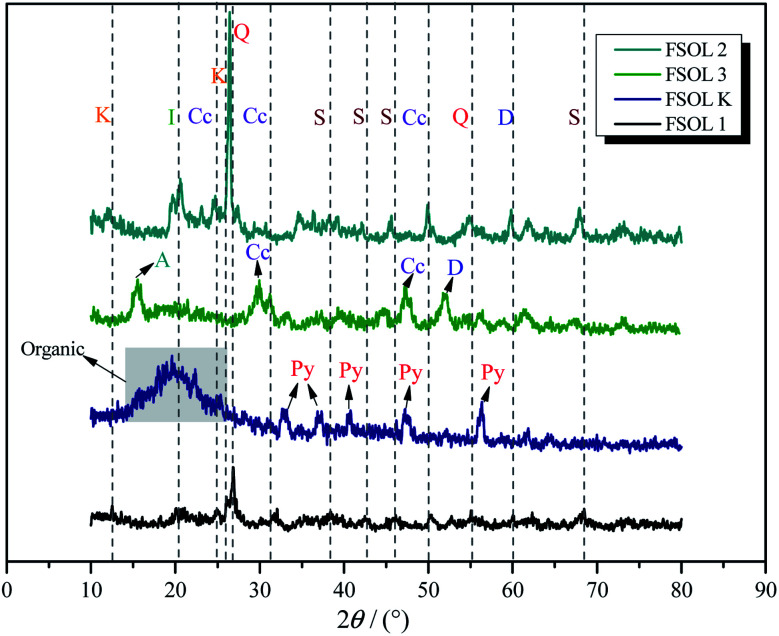
The XRD spectra of the Fushun oil shale four samples. K:kaolinite, Q:quartz, C:calcite, Py:pyrite,I:illite,D:dolomite,F:feldspar.

XRD is widely used for fossil fuel mineral composition. Fig. 2 presents the XRD spectra of Fushun oil shale, carbonate-bearing, silicate-bearing, and kerogen. X-ray diffraction intensities reflect the relative amounts of four minerals. Quartz and calcite were the major minerals identified from original oil shale. Some clay minerals such as montmorillonite and illite were also observed. For the silicate containing samples, the main composition was quartz, illite, and feldspar in the demineralization process of Fushun oil shale using HF and HCL acids. The carbonate-bearing samples primarily consisted of calcite.^30,31^ Also, there was a small amount of pyrite in the kerogen, which is due to the substitution of S atoms on pyrite with C atoms between kerogen and pyrite, resulting in tight cross-linking of organic matter with pyrite. Hence, it is extremely difficult to be removed.^32,33^ Therefore, the acid-washed kerogen samples contained a small amount of pyrite, which also suggests that samples containing different minerals from oil shale can be obtained reliably *via* step-by-step acid-washing. The clay minerals have adsorption energy towards organic matter and strong catalytic activity; therefore, it plays a crucial role in the pyrolysis of organic matter.^34,35^

### 3.2 Thermal decomposition analysis

#### 3.2.1 Heat transfer characteristics

**Fig. 3:**
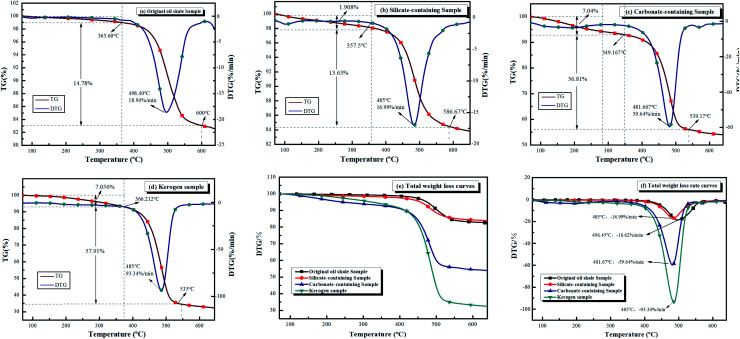
TG and DTG curves of the four samples.

Fig. 2 displays the TG and DTG curves of four samples at optimal heating rate. It can be assumed that the sample pyrolysis reactions were kinetically controlled due to uniform distribution of this sample in the crucible, and hence, there were no significant mass and heat transfer limitations.^36^ Based on the industrial analysis (Table 1) and XRD spectra (Fig. 2), the oil shale samples were high in minerals and low in volatiles. Therefore, the thermal hysteresis and heat transfer limitations were more pronounced with increasing heating rates, shifting the thermogravimetric curve backwards with rising heating rate.^16,37,38^ The mass loss peak on the DTG curve became increasingly wider with the increase in heating rate, which indicates that higher the heating rate, shorter was the pyrolysis time. According to the maximum mass loss rate, the optimal heating rate for the samples of FSOL1, FSOL2, and FSOL3 was 40°C min^−1^, and the optimal heating rate for kerogen was 50°C min^−1^. The pyrolysis of organic matter from oil shale was divided into two main stages. In the first stage, the pyrolysis of kerogen produced solvent-soluble intermediate products of hot bitumen as well as small amount of gaseous products. In this stage, primarily the depolymerization of large heterocyclic compounds and chain opening reactions of long chain molecules occurred. In the second stage, hot bitumen reacted further with bond breaking, ring opening, or aromatization reactions to produce the end products of shale oil, gas, and semi-coke in pyrolysis.^39,40^Fig. 3(a) displays the TG/DTG curves of the original oil shale sample (FSOL1) at 40°C min^−1^. The thermal weight loss process was primarily divided into two stages. The first stage was 0–365.6 °C and the second stage was 395.72–575 °C. Fig. 3(b) shows the TG/DTG curves of the silicate containing sample (FSOL2) at 40°C min^−1^ with two stages, 0–357.5 °C and 357.5–586.6 °C. Fig. 3(c) displays the TG/DTG curves of carbonate-containing sample (FSOL3) at 40°C min^−1^ for two stages, 0–349.2 °C and 349.2–539.17 °C. Fig. 3(d) presents the TG and DTG curves of kerogen (FSOLK) at 50°C min^−1^ with two stages, 0–366.21 °C and 366.21–535 °C. As per Fig. 3(e), the TG curves of FSOL2 and FSOL1 were close and the weight loss of FSOL2 at the later stages of pyrolysis was lower than that of FSOL1, which is due to high content of silicate and presence of adsorption on the pyrolysis products. The weight loss trends of FSOL1, FSOL3, and FSOLK were similar due to the low content of carbonate and the insignificant effect. The DTG weight loss rate curves and TG weight loss curves in Fig. 3(f) presented the same trend. Minerals significantly impacted the pyrolysis end temperature. With the change of minerals, silicate significantly delayed the pyrolysis end temperature, while carbonate slightly delayed the pyrolysis end temperature. The combined effect of both was slightly earlier than the pyrolysis end temperature by the effect of silicate alone. These results indicate that different minerals have different effects on the pyrolysis process. In the following discussion, we further investigate the effect of minerals on the activation energy.

#### 3.2.2 Apparent activation energy estimates according to FWO, Friedman method

Higher *E*_a_ value indicates a higher intermolecular force in the sample and more energy is required to initiate the reaction.^41^ Based on FWO, Friedman method, the kinetic parameters of four samples were calculated. All the measured values of *E*_a_*R*^2^ were in the range of 99.74–99.99%, and there was no significant difference in *E*_a_ between the two methods, which suggests that the estimated results were reasonable (Table 2). By comparing the activation energy of four samples, it was found that FSOL3>FSOLK > FSOL1>FSOL2. The activation energies were 237.22 kJ mol^−1^, 177.60 kJ mol^−1^, 250.45 kJ mol^−1^, and 239.18 kJ mol^−1^. All the activation energies of FSOL2 were lower than those of the other samples. The above conclusions prove that there was a certain inhibitory effect of carbonates, and the adsorption-catalytic promotion of silicates was more prominent. The promotion was greater than the inhibition in Fushun oil shale, which is the same as the conclusions of other researchers.^22,42–44^ Meanwhile, this result provides corresponding theoretical basis for subsequent study of catalytic effect of silicates.

**Table 2:** The mean *E*_a_ (kJ mol^−1^) values of the FSOS1, FSOS2, FSOS3 and FSOSK pyrolysis according to the Friedman and FWO

Sample	Friedman		FWO	
	*E*_a_	*R* ^2^	*E*_a_	*R* ^2^
FS OS1	236.80	0.9974	237.63	0.9984
FS OS2	178.02	0.9978	177.17	0.9986
FS OS3	251.32	0.9972	249.58	0.9987
FS OSK	238.99	0.9976	239.38	0.9979

**Fig. 4:**
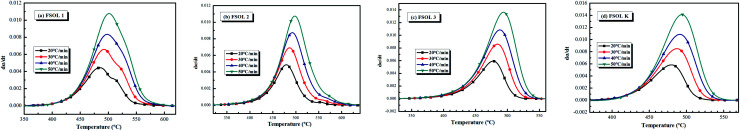
Relationship between time and conversion at different four heating rate.

Fig. 4(a–d) show the conversion rate with temperature, which gradually increased with rise in heating rate; thus, demonstrating the existence of an effect of warming rate on the oil shale pyrolysis.^16^ The conversion rate of FSOLK (Fig. 4d) was largest among the four samples, followed by FSOL3 (Fig. 4c), where an increase in the organic matter content led to an enhancement in the conversion rate. The conversion rates of FSOL2 (Fig. 4b) and FSOL1 (Fig. 6a) were close in magnitude, but it was prominent that the conversion time of FSOL1 was longer; further indicating that the presence of silicate shortened the organic matter conversion time.

### 3.3 Scanning electron microscopy (SEM) analysis

**Fig. 5:**
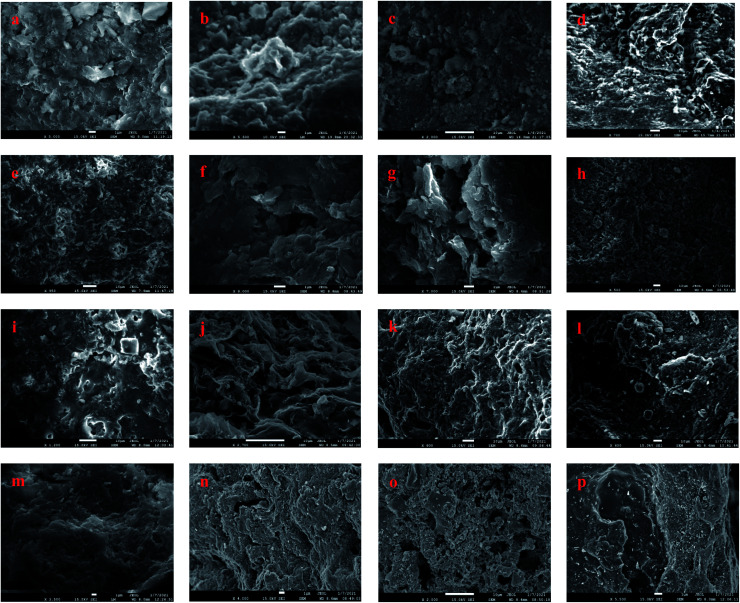
Scanning electron microscope images (a–d): FS-OL1 sample figures; (e–h): FS-OL2 sample figures; (i–l): FS-OL3 sample figures; (m–p): FS-OLK sample figures.

SEM has proven to be an advanced method for direct observation of size, shape, and distribution of organic nanopores.^45^Fig. 5 presents the scanning electron microscopy (SEM) plots of 16 samples, which are the raw samples of FSOL1, FSOL2, FSOL3, and FSOLK and the prepared samples at final temperatures of 400, 470, and 550 °C. The influential role of minerals in the pyrolysis of oil shale by low-temperature dry distillation was primarily due to the pore structure. As per Fig. a, e and i, FSOL1 and FSOL2 were clearly layered and chain-layered, which is due to the crystalline and non-crystalline structure of the clay minerals. As per the industrial analysis (Table 1), the clay minerals dominated. Carbonate is the residue of inorganic matter during the transformation of the original material. During the metamorphosis of organic matter, hydrocarbon inclusions are formed in the pores formed by carbonate; thus, causing some organic matter to be encapsulated by carbonate. As the pyrolysis temperature increased, the layering effect of FSOL2 (e–h) became increasingly prominent. FSOL3 (i–l) preserved the original skeletal shape due to its non-crystalline structure, while FSOL1 (e–h) displayed a less prominent layered shape and retained the common morphology of carbonate-bearing and silicate samples. From figures of FSOLK (m–p) it can be observed that the kerogen is less pore structured during pyrolysis. When the temperature reaches 500 °C, the pyrolysis of the kerogen is in the third stage and a semi-coke structure is formed. From this it can be observed that the semi-coke bonds together to form a stable structure. During the dry distillation pyrolysis of oil shale, pore space is the main pathway for diffusion of shale oil and gas from the kerogen to fracture system.^46,47^ A well-developed organic pore grid has important effects on the porosity, permeability, and hydrocarbon storage. Clay minerals are primarily present in shales as inorganic components with mesopores that account for significant specific surface area. In addition, macropores increase the specific surface area to a certain extent. Therefore, clay minerals in shale have an important influence on the surface area that can adsorb a certain amount of gas.^48,49^ The adsorption capacity of oil shale is controlled by the surface area, size, shape, and volume within the pores and their distribution. Therefore, the parameters of the pore structure are specifically studied in Section 3.4.

### 3.4 Low pressure N_2_ adsorption/desorption measurement

#### 3.4.1 N_2_ adsorption isotherms

**Fig. 6:**
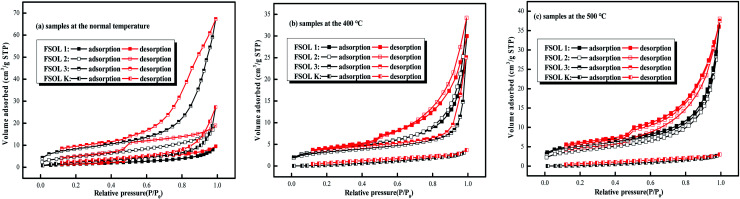
Low-temperature N_2_ adsorption/desorption isotherms.

The adsorption capacity of the samples depends on the pores of organic and inorganic materials, which are favorable sites for adsorption. The shape and hysteresis pattern of low-temperature N_2_ adsorption–desorption isotherms can be effectively used to characterize the pores and morphology of oil shales.^50,51^ The isothermal adsorption/desorption curves of the four samples shown in Fig. 6 were typical of those belonging to type II adsorption/desorption isotherms and H_3_ and H_4_ hysteresis loops. The structural parameters were calculated *via* Barrett–Joyner–Hallenda (BJH) model.^52,53^ The isothermal adsorption and desorption curves were obtained by N_2_ adsorption experiments with relative pressure P/P0 as the horizontal coordinate and adsorption amount as the vertical coordinate. Fig. 6 presents the N_2_ adsorption and desorption curves of FS-OL1, 2, 3, K and the samples at 400 and 500 °C pyrolysis end temperatures. There were three stages in the process. The first stage (0 < *P*/*P*_0_ ≤ 0.4) is the nitrogen adsorption in the low-pressure section, when the adsorption of the gas increased slowly and the adsorption isotherm had a gentle upward shape. The first stage is the monolayer adsorption of nitrogen on the pore surface and the nitrogen adsorption curve presents an inflection point from monolayer to multilayer adsorption.^54^ In the second stage (0.4 < *P*/*P*_0_ ≤ 0.9), the adsorption of the sample increased rapidly and the adsorption isotherm of nitrogen rose rapidly with a hysteresis loop with the increase in relative pressure. In the third stage (*P*/*P*_0_≥0.9), the gas adsorption increased sharply with the rise in relative pressure. The isothermal desorption curve of the sample exhibited a more pronounced desorption hysteresis due to the complex pore structure of the experimental sample. The desorption amount was significantly lower than the adsorption amount, and then, a hysteresis loop appeared. There are significant differences in the developmental morphology and connectivity of the small pores and nitrogen adsorption was not completely closed.^55^

#### 3.4.2 Pore size distribution and pore structure parameters for N_2_ adsorption and desorption

The pore size distribution can be represented by cumulative, incremental, and differential pore volume or surface area *versus* pore diameter curves. By comparing the changes in pore structure during pyrolysis of the four samples, the mechanism of the influence of intrinsic minerals on the pyrolysis of kerogen can then be revealed from a macroscopic perspective. Important information about the structure can be obtained from these curves, including the pore size range, major pore size, and the contribution of different pore size ranges to total pore volume (total surface area). The related calculation methods have been described in the literature.^56–58^Table 3 lists the calculated BET specific surface area, pore volume, and pore size for each of the 12 samples. FSOL2 and FSOL3 demonstrated higher specific surface area and pore volume than those for FSOL1, further indicating that the minerals and organic matter in the Fushun oil shale were closely bonded. The carbonates and silicates closely combined and bonded, resulting in a smaller pore structure. The pore size ranged from 2–50 (nm), suggesting that the pyrolysis of organic matter by minerals was mainly in the mesoporous structure. BET and pore capacity of both FSOL1 and FSOLK samples first increased, and then, decreased with the rise in pyrolysis temperature. This result indicates that the combined effect of silicate and carbonate made the adsorption effect on the pyrolysis products not prominent in the middle stage of pyrolysis. With the rise in temperature, the adsorption effect gradually strengthened leading to the decrease in pore structure. Meanwhile, the BET and pore capacity of both FSOL2 and FSOL3 samples decreased, and then, increased. The final BJH of silicate decreased, which explains stronger adsorption-catalytic effect of silicate and lower activation energy of kerogen in 3.3.1. The final BJH of FSOL3 increased and the effect of carbonate led to higher activation energy of pyrolysis, which may be due to some components in the carbonates. The FSOL2 specific surface and pore volume changes were significantly smaller than those of FSOL3, and this result indicates that the silicate-containing samples are adsorbed, further explaining the reduced activation energy of the silicate-containing samples illustrated in Section 3.2 and demonstrating the catalytic adsorption of silicates. The above conclusions verify that the carbonates in Fushun oil shale had an inhibitory effect and the silicates had a facilitating effect, which is contrary to the conclusions of some other researchers. This may be due to the evolutionary deposition process of oil shale in the samples from different regions and the differences in the structure of organic matter sediments at different arrival times.

**Table 3:** The calculated specific surface area, pore volume and pore size of shale samples

Samples	BET surface area (m^2^ g^−1^)	BJH pore volume(10^−4^cc g^−1^)	BJH average pore diameter(nm)	Cumulative volume pore at different diameter range(%)		
				<2 (nm)	2–50 (nm)	>50 (nm)
FSOL1	5.40	145.14	9.26	1.87	69.91	28.23
FSOL1-400	13.15	448.65	14.32	1.15	55.44	43.41
FSOL1-500	5.29	143.25	9.38	1.74	70.48	27.78
FSOL2	17.78	266.24	6.71	2.03	77.79	20.18
FSOL2-400	12.98	514.71	16.04	0.80	56.90	42.30
FSOL2-500	14.27	571.93	16.93	0.70	54.83	44.47
FSOL3	29.57	1000.13	14.01	0.32	77.99	21.70
FSOL3-400	8.52	369.61	17.35	1.67	35.75	62.58
FSOL3-500	32.37	865.81	14.11	2.04	60.21	37.75
FSOLK	9.12	408.17	16.46	1.03	49.22	34.83
FSOLK-400	11.18	55.97	7.27	3.23	69.26	27.50
FSOLK-500	2.79	45.61	6.94	3.50	76.55	19.94

**Fig. 7:**
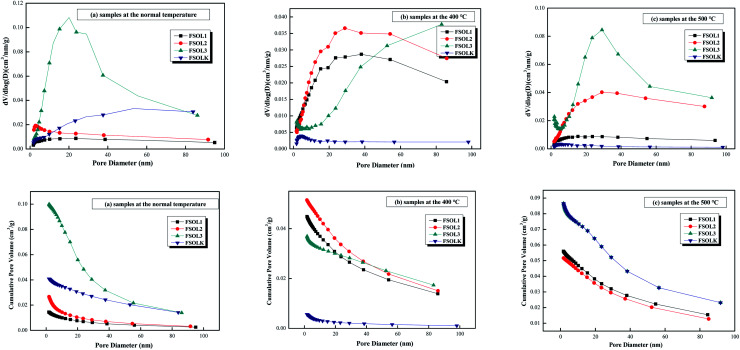
Different presentation of the BJH pore size distributions derived from the N_2_ desorption branch of isotherms for organic-rich shale samples in this study.

The pore size distribution can be expressed as a distribution of pore volume *versus* average pore diameter including differential and cumulative pore volume distribution curves.^58^ The BJH method and N_2_ adsorption data were employed to study the pore size distribution of shale samples. Fig. 7 presents different BJH pore size distributions derived from the N_2_ desorption branch of isotherms for the samples in this study. As per Fig. 7a–c, FSOL1 and FSOL2 were distributed between 1.7–10 nm. The main peak of FSOL3 was distributed between 1.7–40, while that for FSOLK was approximately between 40–60 nm at room temperature. With the rise in temperature, the main peaks of kerogen and original sample tended to be between 1.7–10 nm, which contained carbonate and silicate in the warming process and changed significantly. This phenomenon indicates the existence of the influence of two in the process of organic matter pyrolysis. As per Fig. 7d–f, the pore diameter range of the four samples was primarily concentrated in the range of 1.7–40 nm and the pore size types were small and medium pores. As per previous studies, this pore size is the main structure for oil and gas adsorption.

### 3.5 PY-GC/MS analysis

Py-GC/MS experiments can provide good understanding of the distribution of gaseous products of samples while avoiding the interference of low-temperature pyrolysis products. This is important for the carbon containing pyrolysis products (primarily up to C35^+^) and basically include the organic products generated by the pyrolysis process. These experiments have been widely used in the study of the structure of coal, biomass, and oil shale casein due to rapid breakage of chemical bonds at the end temperature of pyrolysis. This process avoids complex chemical reactions and at the same time avoids the occurrence of secondary reactions to a great extent, which can well impact the basic structure of the samples.^31,59^ Based on the TG pyrolysis curves (Fig. 3a–d), the four samples were completely pyrolyzed before the final temperature of 600 °C; therefore, the thermal cracking experiments were performed on the four samples at the final temperature of 600 °C.

**Fig. 8:**
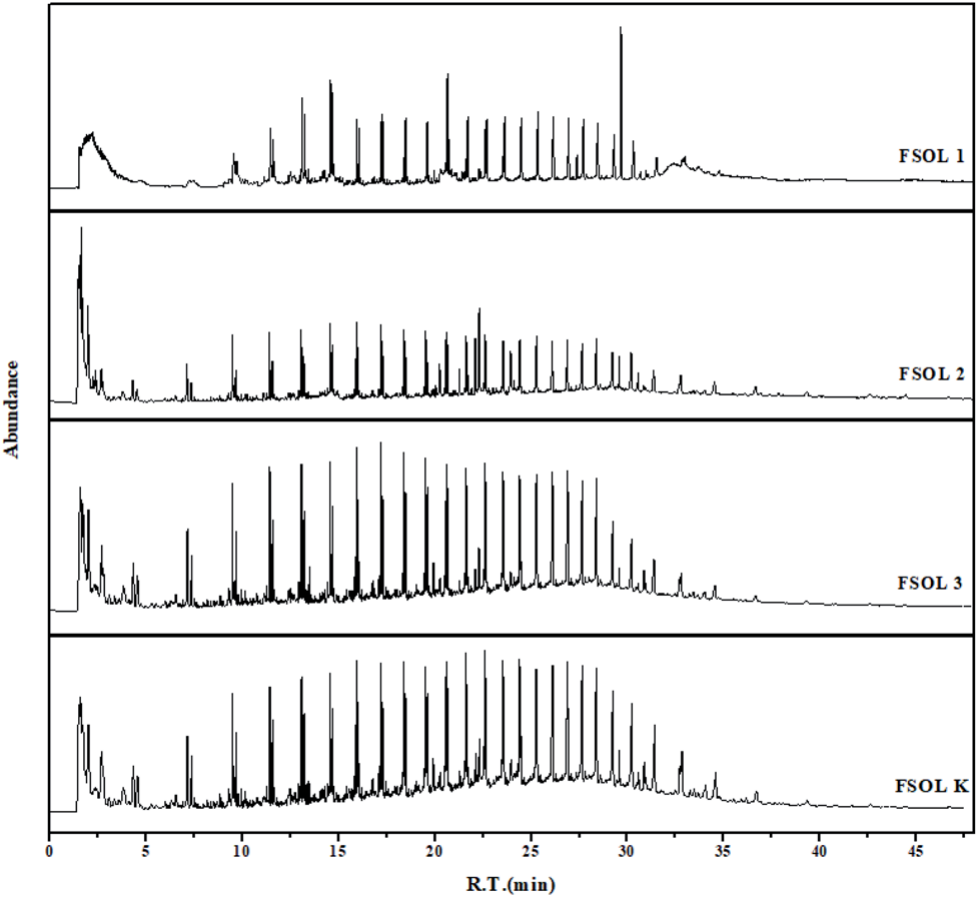
Total ion flow chromatograms of the four samples at 600 °C.

**Fig. 9:**
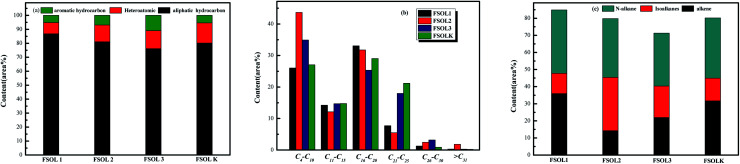
Distribution of four samples pyrolysis products at 600 °C.

Fig. 8 shows the total ion flow chromatograms of FSOL1, FSOL2, FSOL3, and FSOLK with the main identified compounds. As per Fig. 9a, FSOL1 had highest content of aliphatic hydrocarbons and lowest percentage of aromatic hydrocarbon products. FSOLK had highest percentage of heteroatomic compounds, and FSOL3 had highest percentage of fangenine. The percentage of different components of the four samples display that both silicate and carbonates increased the aromatic hydrocarbon products to a certain extent, where silicate increased the aliphatic hydrocarbon content and decreased the production of heteroatomic compounds. Carbonate promoted the production of aromatic hear compounds and inhibited the production of aliphatic hydrocarbon compounds. The combined effect of the two promoted the formation of aliphatic hydrocarbon compounds. The minerals influence the distribution of pyrolysis products during oil shale pyrolysis. As per Fig. 9b, the carbon atomic range of pyrolysis products of all the four samples was between C4 and C25. Among them, both FSOL2 and FSOL3 had largest proportion in the range of C4–C10, indicating that both silicates and carbonates promote the formation of short chains. The analysis of C21–C25 range shows that carbonates lead to the formation of long chains to a certain extent. The joint effect of the two is that the carbon number is larger in the range of C16–C20. As per Fig. 9c, both carbonates and silicates decreased the yield of n-alkanes. The combined effect of both increased the yield of earned alkanes. As per the above analysis, carbonate and silicate and their combined effects impact the components of pyrolysis products to a certain extent. Previous studies have shown that minerals can reduce hydrocarbon yields and promote the cracking of long-chain hydrocarbons to short-chain hydrocarbons, and that minerals can adsorb heavy hydrocarbons, which crack at increasing temperatures to produce light hydrocarbons and carbon residues.^60,61^ The combined carbonate and silicate results indicate that oil shale pyrolysis decreases the amount of long-chain hydrocarbons and increases the amount of short-chain and aromatic hydrocarbons, suggesting that minerals can promote hydrocarbon cracking reactions and aromatization reactions.^62^ Wang *et al.* similarly showed that carbonates can promote shale oil production and inhibit hydrocarbon gas production, while silicates can inhibit shale oil production and promote hydrocarbon gas production, and that both minerals can increase the aromatization of shale oil.^63^ This is the same as the results of this paper. Hence, it can provide a theoretical basis for research on the influence of minerals on the pyrolysis process of organic matter in oil shale.

## Conclusion

This paper applied proximate analysis and XRD, SEM, BET, TG, and Py-GC/MS experiments to investigate the influence of the intrinsic minerals of Fushun oil shale on isothermal and non-isothermal pyrolysis of organic matter from macroscopic and microscopic perspectives. Fushun oil shale ash content was as high as 78.8%, which consisted of silicate, carbonate, quartz, and a small amount of pyrite. The non-isothermal pyrolysis kinetic results indicate that silicates have a pyrolytic catalytic effect on the pyrolysis of organic matter. It was demonstrated by SEM and BET that silicates not only play a catalytic role in the pyrolysis of organic matter, but also have an adsorption effect. It is able to adsorb heavy-value hydrocarbon pyrolysis products during casein pyrolysis, which in turn reduces the yield of hot bitumen. As the activation energy of the carbonate-containing samples is higher than that of the original oil shale samples, this suggests that carbonates have an inhibitory effect on the pyrolysis of the Fushun oil shale. Based on the apparent morphology it can be observed that there is no adsorption of carbonate to hydrocarbon pyrolysis products, but mainly carbonate and metal ions. As per the study of isothermal pyrolysis process, both silicates and carbonates enhanced the aromatic hydrocarbon products to a certain extent, where silicates increased the aliphatic hydrocarbon content and decreased the production of heteroatomic compounds. Carbonates promoted the production of aromatic hear compounds and inhibited the production of aliphatic hydrocarbon compounds. The combined effect of both promoted the formation of aliphatic hydrocarbon compounds. Both carbonates and silicates decreased the yield of n-alkanes, and the combined effect of both increased the yield of n-alkanes.

## Conflicts of interest

There are no conflicts to declare.

## Supplementary Material

RA-012-D2RA02822K-s001
