# Supplementary material for: Study of the effect of in situ minerals on the pyrolysis of oil shale in Fushun, China
Source: RSC Adv. 2022 Jul 13;12(31):20239–50. doi: 10.1039/d2ra02822k (PMC9277521; doi:10.1039/d2ra02822k)
Supplement: RA-012-D2RA02822K-s001 [file RA-012-D2RA02822K-s001.pdf]

Electronic Supplementary Material (ESI) for RSC Advances.
This journal is © The Royal Society of Chemistry 2022

Supporting file S

Table S1 Pyrolysis product parameters of FSOL1 by Py–GC–MS at 600℃.

No.	Compounds(chemical formula)	Percent of peak area	No.	Compounds(chemical formula)	Percent of peak area
1	Propane(C_3H_8)	14.53	48	(Z)-11-Hexadecen-1-ol($C_{16}H_{32}O$)	0.24
2	1,3-Pentadiene(C_5H_8)	7.08	49	1-Hexadecene($C_{16}H_{32}$)	1.55
3	1-Hexene(C_6H_{12})	2.70	50	Hexadecane($C_{15}H_{32}$)	1.28
4	Methylcyclopentadiene(C_6H_8)	0.22	51	(Z)-11-Hexadecen-1-ol($C_{16}H_{32}O$)	0.24
5	2-(Ethylsulfinyl)butane($C_6H_{13}OS$)	0.30	52	1-Hexadecene($C_{16}H_{32}$)	1.48
6	1-Heptene(C_7H_{14})	1.52	53	Hexadecane($C_{15}H_{32}$)	1.27
7	3-Hexanone($C_6H_{12}O$)	0.87	54	Isoheptadecanol (9CI)($C_{17}H_{36}O$)	0.26
8	1,8-Nonadiene(C_9H_{16})	0.33	55	Hexahydrofarnesol($C_{15}H_{32}O$)	0.35
9	3-methylenecyclohexene(C_7H_{10})	0.26	56	Pentadecanoic acid($C_{15}H_{30}O_2$)	0.32
10	(Z)-3-Hepten-1-ol($C_7H_{14}O$)	0.31	57	(Z)-11-Hexadecen-1-ol($C_{16}H_{32}O$)	0.25
11	Toluene(C_7H_8)	1.44	58	(3Z)-3-Hexadecene($C_{16}H_{32}$)	1.45
12	1-Octene(C_8H_{16})	1.38	59	Hexadecane($C_{16}H_{34}$)	1.19
13	1-octane(C_8H_{18})	1.01	60	3-Octadecene($C_{18}H_{36}$)	1.39
14	Isopropylidenecyclopentane(C_8H_{14})	0.25	61	Pentadecane($C_{15}H_{32}$)	1.20
15	2,6-Dimethyl-1-heptene(C_9H_{18})	0.38	62	9-Hexadecenoic acid($C_{16}H_{30}O_2$)	0.35
16	Ethylbenzene(C_8H_{10})	0.25	63	Palmitic acid($C_{16}H_{32}O_2$)	0.87
17	1,4-Xylene(C_8H_{10})	0.38	64	ethanol,2-[(9Z)-9-octadecenyloxy]- [illegible]	0.22
18	1-Nonene(C_9H_{18})	1.84	65	(E)-3-Icosene($C_{20}H_{40}$)	1.33
19	n-Nonane(C_9H_{20})	0.92	66	Hexadecane($C_{16}H_{34}$)	1.18
20	2,6-Dimethyl-3-heptene(C_9H_{18})	0.21	67	(E)-3-Icosene($C_{20}H_{40}$)	1.15
21	1-ethyl-3-methyl-Benzene(C_9H_{12})	0.24	68	Pentadecane($C_{15}H_{32}$)	1.16
22	Phenol(C_6H_6O)	0.29	69	9-Hexadecenoic acid($C_{16}H_{30}O_2$)	0.30
23	1,9-Decadiene($C_{10}H_{18}$)	0.30	70	(E)-3-Icosene($C_{20}H_{40}$)	1.03
24	1-Decene($C_{10}H_{20}$)	1.69	71	Hexadecane($C_{16}H_{34}$)	1.02
25	Decane($C_{10}H_{22}$)	0.79	72	ethanol,2-[(9Z)-9-octadecenyloxy]-($C_{20}H_{40}O_2$)	0.20
26	3,7,11-Trimethyl-1-dodecanol($C_{15}H_{32}O$)	0.21	73	(E)-3-Icosene($C_{20}H_{40}$)	1.26
27	2-Methylphenol(C_7H_8O)	0.29	74	Heptadecane($C_{17}H_{36}$)	1.58
28	3-Hydroxytoluene(C_7H_8O)	0.35	75	1-Heneicosanol($C_{21}H_{44}O$)	1.27
29	Octylcyclopropane($C_{11}H_{22}$)	1.72	76	Hexadecane($C_{16}H_{34}$)	1.33
30	Undecane($C_{11}H_{24}$)	0.93	77	(E)-3-Icosene($C_{20}H_{40}$)	1.25
31	2,3-Dimethylphenol($C_8H_{10}O$)	0.21	78	Heneicosane($C_{21}H_{44}$)	1.32

32	2-Methylene-5-isopropenylcyclohexanol($C_{10}H_{16}O$)	0.21	79	1-Heneicosanol,1-formate($C_{22}H_{44}O_2$)	1.14
33	1,11-Dodecadiene($C_{12}H_{22}$)	0.24	80	2-Methylheptadecane($C_{18}H_{38}$)	1.28
34	(E)-2-Dodecene($C_{12}H_{24}$)	1.74	81	19-Methyleicosane($C_{21}H_{44}$)	2.68
35	Dodecane($C_{12}H_{26}$)	1.00	82	17-Pentatriacontene($C_{35}H_{70}$)	0.25
36	3-DODECENE($C_{12}H_{24}$)	0.21	83	17-Pentatriacontene($C_{35}H_{70}$)	0.22
37	2,6-Dimethylundecane($C_{13}H_{28}$)	0.22	84	18-Methylnonadecane($C_{20}H_{42}$)	2.15
38	1,10-Undecadiene($C_{11}H_{20}$)	0.20	85	trans-Squalene($C_{30}H_{50}$)	0.52
39	1-Undecene($C_{11}H_{22}$)	1.52	86	18-Methylnonadecane($C_{20}H_{42}$)	2.01
40	Tridecane($C_{13}H_{28}$)	1.01	87	Cholesterilene($C_{27}H_{44}$)	0.54
41	1,12-Tridecadiene($C_{13}H_{24}$)	0.26	88	2-(7-Heptadecynyloxy)tetrahydro-2H-pyran($C_{22}H_{40}O_2$)	0.47
42	(Z)-3-Tetradecene($C_{14}H_{28}$)	1.67	89	2,6,10-Trimethyltetradecane($C_{17}H_{36}$)	1.47
43	Pentadecane($C_{15}H_{32}$)	1.12	90	Heneicosane($C_{21}H_{44}$)	0.84
44	2,7,10-Trimethyldodecane($C_{15}H_{32}$)	0.21	91	2,6,10-Trimethyltetradecane($C_{17}H_{36}$)	0.58
45	(Z)-11-Hexadecen-1-ol($C_{16}H_{32}O$)	0.24	92	Tetracosane, 11-decyl-($C_{34}H_{70}$)	0.31
46	1-Hexadecene($C_{16}H_{32}$)	1.67	93	3-Ethyl-5-(2-ethylbutyl)octadecane($C_{26}H_{54}$)	0.20
47	Pentadecane($C_{15}H_{32}$)	1.30			

Table S2 Pyrolysis product parameters of FSOL2 by Py–GC–MS at 600℃.

No.	Compounds(chemical formula)	Percent of peak area	No.	Compounds(chemical formula)	Percent of peak area
1	2-Butene（C_4H_8）	15.56	46	Myristic acid（$C_{14}H_{28}O_2$）	1.04
2	1,4-Pentadiene（C_5H_8）	8.75	47	1-Octadecyne（$C_{18}H_{34}$）	0.20
3	1-Hexene（C_6H_{12}）	3.52	48	1-Hexadecene（$C_{16}H_{32}$）	1.14
4	1,4-cyclohexadiene（C_6H_8）	0.43	49	Hexadecane（$C_{16}H_{34}$）	0.92
5	Benzene（C_6H_6）	1.04	50	Palmitic acid（$C_{16}H_{32}O_2$）	0.69
6	1-Heptene（C_7H_{14}）	1.38	51	3-Octadecene（$C_{18}H_{36}$）	1.09
7	3-Hexanone（$C_6H_{12}O$）	0.77	52	Hexadecane（$C_{16}H_{34}$）	0.89
8	3-Hexanone（C_7H_{10}）	0.30	53	9-Hexadecenoic acid,(9Z)-（$C_{16}H_{30}O_2$）	0.32
9	3-Hepten-1-ol, (3E)-（$C_7H_{14}O$）	0.41	54	Palmitic acid（$C_{16}H_{32}O_2$）	0.21
10	Toluene（C_7H_8）	1.04	55	Palmitic acid（$C_{16}H_{32}O_2$）	1.72
11	1-Octene（C_8H_{16}）	1.23	56	Palmitic acid（$C_{16}H_{32}O_2$）	2.80
12	1-octane（C_8H_{18}）	0.81	57	1-Hexadecene（$C_{16}H_{32}$）	1.00

13	Cyclohexane,1-methyl-2-methylene-（C_8H_{14}）	0.26	58	Dodecane,2,7,10-trimethyl-（$C_{15}H_{32}$）	0.85
14	Ethylbenzene（C_8H_{10}）	0.24	59	Oleic acid（$C_{18}H_{34}O_2$）	0.20
15	Ethylbenzene（C_8H_{10}）	0.36	60	3-Eicosene,(E)-（$C_{20}H_{40}$）	0.84
16	1-Nonene（C_9H_{18}）	1.48	61	Dodecane,2,7,10-trimethyl-（$C_{15}H_{32}$）	0.91
17	n-Nonane（C_9H_{20}）	0.69	62	Oleic acid（$C_{18}H_{34}O_2$）	1.86
18	1-ethyl-3-methyl-Benzene（C_9H_{12}）	0.22	63	Stearic acid（$C_{18}H_{36}O_2$）	0.28
19	Dihydromyrcene（$C_{10}H_{18}$）	0.34	64	(E)-5-Icosene（$C_{20}H_{40}$）	0.97
20	1-Decene（$C_{10}H_{20}$）	1.39	65	Hexadecane（$C_{16}H_{34}$）	1.03
21	1-ethyl-4-methyl-Benzene（C_9H_{12}）	0.29	66	Hexadecane（$C_{16}H_{34}$）	1.15
22	Decane（$C_{10}H_{22}$）	0.68	67	3-Eicosene,(E)-（$C_{20}H_{40}$）	0.95
23	2-propyl-1-Heptanol（$C_{10}H_{22}O$）	0.20	68	Heneicosane（$C_{21}H_{44}$）	0.95
24	m-Cresol（C_7H_8O）	0.36	69	Heneicosane（$C_{21}H_{44}$）	0.89
25	1-Decene（$C_{10}H_{20}$）	1.37	70	3-Eicosene,(E)-（$C_{20}H_{40}$）	0.94
26	Undecane（$C_{11}H_{24}$）	0.73	71	Heneicosane（$C_{21}H_{44}$）	0.96
27	3,4-Dimethylphenol（$C_8H_{10}O$）	0.22	72	Octadecane, 2-methyl-（$C_{19}H_{40}$）	2.26
28	p-isopropyltoluene（$C_{10}H_{14}$）	0.34	73	17-Pentatriacontene（$C_{35}H_{70}$）	0.29
29	1-Dodecene（$C_{12}H_{24}$）	1.41	74	Eicosane, 2-methyl-（$C_{21}H_{44}$）	2.07
30	Dodecane（$C_{12}H_{26}$）	0.82	75	(E,E,E,E)-Squalene（$C_{30}H_{50}$）	0.97
31	1-Ethyl-1-methylindane（$C_{12}H_{16}$）	0.22	76	Octadecane, 2-methyl-（$C_{19}H_{40}$）	1.89
32	1-Dodecene（$C_{12}H_{24}$）	1.27	77	Cholesteryl myristate（$C_{41}H_{72}O_2$）	0.85
33	Tridecane（$C_{13}H_{28}$）	0.88	78	2-Methylnonadecane（$C_{20}H_{42}$）	1.54
34	1-pentyl-2-propyl-Cyclopentane（$C_{13}H_{26}$）	0.19	79	2-Methylnonadecane（$C_{20}H_{42}$）	1.30
35	o-phthalic anhydride（$C_8H_4O_3$）	0.20	80	9-Hexadecenoic acid,tetradecyl ester, (9Z)-（$C_{30}H_{58}O_2$）	0.30
36	1,12-Tridecadiene（$C_{13}H_{24}$）	0.24	81	2-Methylnonadecane（$C_{20}H_{42}$）	1.20
37	3-Tetradecene,(Z)-（$C_{14}H_{28}$）	1.33	82	Octadecane, 2-methyl-（$C_{19}H_{40}$）	1.02
38	Tetradecane（$C_{14}H_{30}$）	0.85	83	9-Hexadecenoic acid,eicosyl ester, (9Z)-（$C_{36}H_{70}O_2$）	0.24
39	1-Hexadecene（$C_{16}H_{32}$）	1.32	84	9-Hexadecenoic acid,eicosyl ester, (9Z)-（$C_{36}H_{70}O_2$）	0.23
40	Pentadecane（$C_{15}H_{32}$）	1.01	85	Eicosane, 2-methyl-（$C_{21}H_{44}$）	0.63
41	7-Hexadecene, (7Z)-（$C_{16}H_{32}$）	1.22	86	Heneicosane,11-(1-ethylpropyl)-（$C_{26}H_{54}$）	0.66
42	Dodecane,2,6,11-trimethyl-（$C_{15}H_{32}$）	0.98	87	9-Hexadecenoic acid,tetradecyl ester, (9Z)-（C30H58O2）	0.22

43	3-Octadecene（$C_{18}H_{36}$）	1.13	88	9-Hexadecenoic acid,eicosyl ester, (9Z)- [illegible]	0.49
44	Hexadecane（$C_{16}H_{34}$）	0.97	89	Octadecane,3-ethyl-5-(2-ethylbutyl)-（$C_{26}H_{54}$）	0.32
45	5-Methyl-1-undecene（$C_{12}H_{24}$）	0.26	90		

Table S3 Pyrolysis product parameters of FSOL3 by Py–GC–MS at 600℃.

No.	Compounds(chemical formula)	Percent of peak area	No.	Compounds(chemical formula)	Percent of peak area
1	2-Butene（C_4H_8）	6.95	47	1,14-Tetradecanediol（$C_{14}H_{30}O_2$）	0.28
2	Cyclopentene（C_5H_8）	0.97	48	1-Hexadecene（$C_{16}H_{32}$）	1.84
3	DIHYDRO-3,5-DIMETHYL-2(3H)-FURANONE（$C_6H_{10}O_2$）	4.45	49	Hexadecane（$C_{16}H_{34}$）	1.45
4	1-Hexene（C_6H_{12}）	3.08	50	1,14-Tetradecanediol（$C_{14}H_{30}O_2$）	0.27
5	1,3-Cyclohexadiene（C_6H_8）	0.31	51	1-Hexadecene（$C_{16}H_{32}$）	1.73
6	1,3-Pentadiene,4-methyl-（C_6H_{10}）	0.30	52	Heptadecane（$C_{17}H_{36}$）	1.34
7	1-Heptene（C_7H_{14}）	1.83	53	2-hexyl-1-Decanol（$C_{16}H_{34}O$）	0.24
8	3-Hexanone（$C_6H_{12}O$）	1.10	54	1-Dodecanol,3,7,11-trimethyl-（$C_{15}H_{32}O$）	0.53
9	4-Nonenal, (4E)-（$C_9H_{16}O$）	0.36	55	Myristic acid（$C_{14}H_{28}O_2$）	0.40
10	3-methylenecyclohexene（C_7H_{10}）	0.31	56	1,15-Pentadecanediol（$C_{15}H_{32}O_2$）	0.25
11	3-Hepten-1-ol, (3E)-（$C_7H_{14}O$）	0.25	57	1-Hexadecene（$C_{16}H_{32}$）	1.64
12	Toluene（C_7H_8）	1.18	58	Eicosane（$C_{20}H_{42}$）	1.29
13	1-Octene（C_8H_{16}）	1.57	59	Pentadecanoic acid（$C_{15}H_{30}O_2$）	0.32
14	1-octane（C_8H_{18}）	1.28	60	1,15-Pentadecanediol（$C_{15}H_{32}O_2$）	0.24
15	1-Methyl-2-methenylcyclohexane（C_8H_{14}）	0.31	61	(E)-5-Icosene（$C_{20}H_{40}$）	1.60
16	1-Heptene,2,6-dimethyl-（C_9H_{18}）	0.27	62	Eicosane（$C_{20}H_{42}$）	1.28
17	Cyclopentene,1,2,4,4-tetramethyl-（C_9H_{16}）	0.34	63	9-Hexadecenoic acid（$C_{16}H_{30}O_2$）	0.47
18	1,3-Xylene（C_8H_{10}）	0.56	64	Palmitic acid（$C_{16}H_{32}O_2$）	0.80
19	1-Nonene（C_9H_{18}）	2.00	65	1-Octadecene（$C_{18}H_{36}$）	1.48
20	n-Nonane（C_9H_{20}）	1.14	66	Eicosane（$C_{20}H_{42}$）	1.29
21	1-ethyl-3-methyl-Benzene（C_9H_{12}）	0.30	67	3-Eicosene,(E)-（$C_{20}H_{40}$）	1.28
22	1,9-Decadiene（$C_{10}H_{18}$）	0.37	68	Heptadecane（$C_{17}H_{36}$）	1.28
23	1-Decene（$C_{10}H_{20}$）	2.35	69	TRANS-13-OCTADECENOIC ACID（$C_{18}H_{34}O_2$）	0.58
24	Decane（$C_{10}H_{22}$）	1.06	70	1-tricosene（$C_{23}H_{46}$）	1.24
25	2-propyl-1-Heptanol（$C_{10}H_{22}O$）	0.28	71	Heneicosane（$C_{21}H_{44}$）	1.21
26	o-Cresol（C_7H_8O）	0.25	72	1,19-Eicosadiene（$C_{20}H_{38}$）	0.24
27	m-Cresol（C_7H_8O）	0.36	73	3-Eicosene,(E)-（$C_{20}H_{40}$）	1.57

28	1-Undecene（$C_{11}H_{22}$）	1.93	74	Heptadecane（$C_{17}H_{36}$）	1.63
29	Undecane（C_{11H24}）	1.15	75	1-TETRACOSANOL（$C_{24}H_{50}O$）	1.66
30	3,4-Dimethylphenol（$C_8H_{10}O$）	0.27	76	Tetracosane（$C_{24}H_{50}$）	1.58
31	1H-Indene, 1-methyl-（$C_{10}H_{10}$）	0.24	77	1-TETRACOSANOL（$C_{24}H_{50}O$）	1.42
32	1,10-Undecadiene（$C_{11}H_{20}$）	0.28	78	1-Heptacosanol（$C_{27}H_{56}O$）	1.49
33	1-Dodecene（$C_{12}H_{24}$）	1.92	79	Tetracosane（$C_{24}H_{50}$）	1.37
34	Dodecane（$C_{12}H_{26}$）	1.19	80	Eicosane, 2-methyl-（$C_{21}H_{44}$）	2.83
35	Undecane, 2,6-dimethyl-（$C_{13}H_{28}$）	0.26	81	17-Pentatriacontene（$C_{35}H_{70}$）	0.29
36	2,4-Dimethylbenzaldehyde（$C_9H_{10}O$）	0.50	82	9-hexacosene（$C_{26}H_{52}$）	0.23
37	Octane, 2,6-dimethyl-（$C_{10}H_{22}$）	0.35	83	2-Methyleicosane（$C_{21}H_{44}$）	2.28
38	1-Tridecanol（$C_{13}H_{28}O$）	1.71	84	(E,E,E,E)-Squalene（$C_{30}H_{50}$）	0.38
39	Tetradecane（$C_{14}H_{30}$）	1.14	85	2-Methyleicosane（$C_{21}H_{44}$）	1.94
40	11-Hexadecen-1-ol,(11Z)-（$C_{16}H_{32}O$）	0.27	86	Cholesta-3,5-diene（$C_{27}H_{44}$）	0.26
41	3-Tetradecene,(Z)-（$C_{14}H_{28}$）	1.89	87	Heptadecane,2,6,10,15-tetramethyl-（$C_{21}H_{44}$）	1.54
42	Tetradecane（$C_{14}H_{30}$）	1.23	88	Tetracosane（$C_{24}H_{50}$）	1.10
43	Dodecane,2,6,10-trimethyl-（$C_{15}H_{32}$）	0.25	89	17aH-Norhopane（$C_{29}H_{50}$）	0.32
44	1-Octadecyne（$C_{18}H_{34}$）	0.33	90	Heptadecane,2,6,10,15-tetramethyl（$C_{21}H_{44}$）	0.91
45	1-Hexadecene（$C_{16}H_{32}$）	1.92	91	Nonadecane, 2-methyl-（$C_{25}H_{52}$）	0.51
46	Pentadecane（$C_{15}H_{32}$）	1.43	92	Tetracosane, 11-decyl-（$C_{34}H_{70}$）	0.28

Table S4 Pyrolysis product parameters of FSOLK by Py–GC–MS at 600℃.

No.	Compounds(chemical formula)	Percent of peak area	No.	Compounds(chemical formula)	Percent of peak area
1	2-Butene（C_4H_8）	6.64	48	(3Z)-3-Hexadecene（$C_{16}H_{32}$）	1.52
2	1,3-Pentadiene, (3E)-（C_5H_8）	6.05	49	Hexadecane（$C_{16}H_{34}$）	1.32
3	1-Hexene（C_6H_{12}）	3.76	50	Dodecane,2,6,10-trimethyl-（$C_{15}H_{32}$）	0.34
4	1-Methylcyclopentene（C_6H_{10}）	1.09	51	1-Hexadecene（$C_{16}H_{32}$）	1.37
5	1-Heptene（C_7H_{14}）	2.91	52	Heptadecane（$C_{17}H_{36}$）	1.02
6	Dihydro-4-methyl-2H-pyran（$C_6H_{10}O$）	0.27	53	1-Dodecanol,3,7,11-trimethyl-（$C_{15}H_{32}O$）	0.54

7	2-Methyl-1,3,5-hexatriene（C_7H_{10}）	0.25	54	TRANS-13-OCTADECENOIC ACID（$C_{18}H_{34}O_2$）	0.23
8	Toluene（C_7H_8）	1.01	55	Myristic acid（$C_{14}H_{28}O_2$）	0.34
9	1-Octene（C_8H_{16}）	1.41	56	1-Hexadecene（$C_{16}H_{32}$）	1.35
10	1-octane（C_8H_{18}）	1.27	57	Eicosane（$C_{20}H_{42}$）	0.95
11	Cyclohexene,1,2-dimethyl-（C_8H_{14}）	0.25	58	Palmitic acid（$C_{16}H_{32}O_2$）	0.22
12	1-Heptene,2,6-dimethyl-（C_9H_{18}）	0.29	59	Octadecanal（$C_{18}H_{36}O$）	0.25
13	Ethylbenzene（C_8H_{10}）	0.29	60	Nonadecanol（$C_{19}H_{40}O$）	1.37
14	1,4-Xylene（C_8H_{10}）	0.38	61	Eicosane（$C_{20}H_{42}$）	1.03
15	(Z)-2-Nonene（C_9H_{18}）	1.72	62	9-Hexadecenoic acid,(9Z)-（$C_{16}H_{30}O_2$）	0.46
16	n-Nonane Nonane nonyl hydride（C_9H_{20}）	1.06	63	Palmitic acid（$C_{16}H_{32}O_2$）	0.75
17	1-ethyl-3-methyl-Benzene（C_9H_{12}）	0.31	64	1-Octadecene（$C_{18}H_{36}$）	1.31
18	1,9-Decadiene（$C_{10}H_{18}$）	0.30	65	Eicosane（$C_{20}H_{42}$）	1.08
19	1-Decene（$C_{10}H_{20}$）	2.01	66	3-Eicosene,(E)-（$C_{20}H_{40}$）	1.17
20	n-Decane（$C_{10}H_{22}$）	0.95	67	Eicosane（$C_{20}H_{42}$）	1.13
21	2-propyl-1-Heptanol（$C_{10}H_{22}O$）	0.27	68	TRANS-13-OCTADECENOIC ACID（$C_{18}H_{34}O_2$）	0.61
22	1-ethyl-3-methyl-Benzene（C_9H_{12}）	0.23	69	Eicosanoic acid（$C_{20}H_{40}O_2$）	0.23
23	Cyclopentane, isopentyl-（$C_{10}H_{20}$）	0.27	70	1-Heneicosanol,1-formate（$C_{22}H_{44}O_2$）	1.53
24	Styrene, 3,4-dimethyl-（$C_{10}H_{12}$）	0.23	71	Heneicosane（$C_{21}H_{44}$）	1.46
25	1-Undecene（$C_{11}H_{22}$）	1.62	72	Hexadecane,1,1-bis(dodecyloxy)-（$C_{40}H_{82}O_2$）	0.22
26	Undecane（$C_{11}H_{24}$）	1.05	73	1,19-Eicosadiene（$C_{20}H_{38}$）	0.26
27	(E)-4-Undecene（$C_{11}H_{22}$）	0.23	74	1-Heneicosanol,1-formate（$C_{22}H_{44}O_2$）	1.43
28	1H-Indene, 1-methyl-（$C_{10}H_{10}$）	0.28	75	Heneicosane（$C_{21}H_{44}$）	1.52
29	Benzenethiol, dimethyl-（$C_8H_{10}S$）	0.21	76	1-TETRACOSANOL（$C_{24}H_{50}O$）	0.22
30	1,10-Undecadiene（$C_{11}H_{20}$）	0.23	77	1-TETRACOSANOL（$C_{24}H_{50}O$）	1.60
31	1-Dodecene（$C_{12}H_{24}$）	1.57	78	Tetracosane（$C_{24}H_{50}$）	1.33
32	Dodecane（$C_{12}H_{26}$）	1.07	79	1-TETRACOSANOL（$C_{24}H_{50}O$）	1.61
33	3-DODECENE（$C_{12}H_{24}$）	0.25	80	Heneicosane（$C_{21}H_{44}$）	1.33
34	Undecane, 2,6-dimethyl-（$C_{13}H_{28}$）	0.27	81	(E)-5-Icosene（$C_{20}H_{40}$）	0.23
35	Tetrahydro-2-[(1-methyl-4-phenyl-2-butynyl)oxy]-2H-pyran	0.22	82	2-octyl-1-Dodecanol（$C_{20}H_{42}O$）	1.56
36	11-Hexadecen-1-ol,(11Z)-（$C_{16}H_{32}O$）	0.24	83	Tetracosane（$C_{24}H_{50}$）	1.29
37	1-Undecene（$C_{11}H_{22}$）	1.49	84	Eicosane（$C_{20}H_{42}$）	3.08
38	Dodecane（$C_{12}H_{26}$）	1.04	85	Eicosane（$C_{20}H_{42}$）	2.85
39	1-Undecanol, 2-methyl-（$C_{12}H_{26}O$）	0.23	86	(E,E,E,E)-Squalene（$C_{30}H_{50}$）	0.51

40	1,11-Dodecadiene（$C_{12}H_{22}$）	0.23	87	Tetracosane（$C_{24}H_{50}$）	2.96
41	1-Tetradecene（$C_{14}H_{28}$）	1.61	88	Cholesta-3,5-diene（$C_{27}H_{44}$）	0.36
42	Tetradecane（$C_{14}H_{30}$）	1.19	89	2-Methyleicosane（$C_{21}H_{44}$）	2.30
43	2-Hexyl-1-octanol（$C_{14}H_{30}O$）	0.22	90	Heneicosane（$C_{21}H_{44}$）	1.47
44	Hexadecane（$C_{16}H_{34}$）	0.24	91	2-Methyleicosane（$C_{21}H_{44}$）	1.15
45	11-Hexadecen-1-ol,(11Z)-（$C_{16}H_{32}O$）	0.24	92	Nonadecane, 2-methyl-（$C_{20}H_{42}$）	0.68
46	(Z)-7-Hexadecene（$C_{16}H_{32}$）	1.64	93	Nonadecane, 2-methyl-（$C_{20}H_{42}$）	0.39
47	Dodecane,2,6,11-trimethyl-（$C_{15}H_{32}$）	1.24	94	Nonadecane, 2-methyl-（$C_{20}H_{42}$）	0.24
